# Feeding Alginate-Coated Liquid Metal Nanodroplets to Silkworms for Highly Stretchable Silk Fibers

**DOI:** 10.3390/nano12071177

**Published:** 2022-04-01

**Authors:** Zhong-Feng Gao, Lin-Lin Zheng, Wen-Long Fu, Lei Zhang, Jin-Ze Li, Pu Chen

**Affiliations:** 1 Advanced Materials Institute, Shandong Academy of Sciences, Qilu University of Technology, Jinan 250014, China; f58445646@163.com; 2 College of Chemistry and Chemical Engineering, Linyi University, Linyi 276005, China; zhenglinlinya@163.com (L.-L.Z.); ljz9711@163.com (J.-Z.L.); 3 Department of Chemical Engineering and Waterloo Institute for Nanotechnology, University of Waterloo, 200 University Avenue West, Waterloo, ON N2L 3G1, Canada; l78zhang@uwaterloo.ca

**Keywords:** silkworm silk, liquid metal, sodium alginate, silk fibroin, functional materials

## Abstract

In this study, we fed the larval of Bombyx mori silkworms with nanodroplets of liquid metal (LM) coated with microgels of marine polysaccharides to obtain stretchable silk. Alginate-coated liquid metal nanodroplets (LM@NaAlg) were prepared with significant chemical stability and biocompatibility. This study demonstrates how the fed LM@NaAlg acts on the as-spun silk fiber. We also conducted a series of characterizations and steered molecular dynamics simulations, which showed that the LM@NaAlg additions impede the conformation transition of silk fibroins from the random coil and α-helix to the β-sheet by the formation of hydrogen bonds between LM@NaAlg and the silk fibroins, thus enhancing the elongation at the breakpoints in addition to the tensile properties. The intrinsically highly stretchable silk showed outstanding mechanical properties compared with regular silk due to its 814 MPa breaking strength and a breaking elongation of up to 70%—the highest reported performance so far. We expect that the proposed method can expand the fabrication of multi-functional silks.

## 1. Introduction

Silk has been used as a natural filament fiber for hundreds of years. It has been widely used in the biomedical field, textile industry, and even as an engineering material due to its favorable biocompatibility, controllable biodegradability, lustrous appearance, and excellent mechanical properties [1,2,3,4]. Various functional components, such as fluorescent proteins [5], rare-earth upconverting phosphors [6], antimicrobial agents [7,8], metal ions [9], metal and semiconductor nanodroplets [10,11], and graphene quantum dots [12,13,14], have been used to intrinsically produce functionalized silks. Extrinsic and intrinsic functionalization approaches have been used to improve the performance of silk. Traditional extrinsic functionalization methods add modifiers to the surface of silk [15,16,17,18]—which inevitably requires the use of toxic chemicals—or re-spin the structure of regenerated silk using additives [19,20]. Many researchers have been devoted to finding an alternative way to incorporate external substances into silkworm silk to enhance its mechanical properties. There are mainly three methods used for improving the properties of silk fibers: gene overexpression [21,22], feeding [23,24,25], and injecting [26,27]. Lizuka et al. reported that silkworms used for the mass production of three colors (green, red, and orange) of fluorescent silk can be generated using a vector originating from the fibroin H chain gene [28]. Although significant performances were realized through genetic alterations, the synthesis procedures are complicated and very costly. In comparison, the in vivo uptake, feeding, and injecting approaches are much easier and cheaper. Ma et al. successfully increased the toughness modulus of silks by the intravascular injection of albumin bovine (BSA)-stabilized gold nanoclusters [29]. Compared with injections, which are lethal and not suitable for large-scale production, feeding specific diets to silkworms is among the most common approaches to functionalizing silk fibers due to its convenient and green properties. Several groups have acquired enhanced stretchable silks by feeding additives, including amino acids [30], dyes [31,32], and nanomaterials [12,23,25], to silkworms. For example, Wang et al. revealed that high-strength silks can be directly obtained by feeding silkworms with graphene nanosheets [25]. Wu et al. characterized the impact of the mechanical properties of the resulting silk fibers from silkworms fed with different nanoparticles (Cu, Fe, and TiO_2_), and the obtained Cu-containing silk fibers exhibited a good tensile strength of 360 MPa and reached a strain of 38% [25]. Although the reported nanomaterial feeding methods increased the silk toughness, the rigid intrinsic property of the used nanomaterials restricted the stretchability extent. Thus, further improving the stretchability performance of silk remains an essential research area.

Liquid metal possesses both “liquid” and “metallic” properties [33], and it is a promising material for soft bioelectronics due to its excellent conductivity, stretchability, super compliance, low cost, and environmental processing technology. Compared with traditional stiff materials, the emerging gallium-based liquid metals with the properties of low Young’s moduli (1–10 Pa), which are infinitely deformable in principle, have drawn a great deal of attention as ideal candidates for fabricating highly stretchable devices [34,35]. However, to our best knowledge, feeding silkworms with liquid metal has not been explored.

In this study, directly from the second day of their fifth instar, we fed Bombyx mori silkworms with diets containing nanodroplets of alginate-coated liquid metal (LM@NaAlg), which significantly improved the tensile property of silk more efficiently than that of the reported rigid materials, to obtain intrinsically reinforced silkworm silk fibers. The alloys of Ga, In, and Sn were used as liquid metals in this work. The conducted cytotoxic experiment indicated that the LM@NaAlg nanodroplets had no obvious negative effects on the growth status of silkworms. Also, the silk properties were characterized by analyzing their thermal stability, and the silk structures were examined using a scanning electron microscope (SEM). We studied the dissolved silk fibers using Fourier transform infrared (FTIR) spectra and X-ray diffraction (XRD), and it was confirmed that the incorporation of parts of LM@NaAlg into the as-obtained silk fibers was successful. The evidence for the mesophase in silkworm silks was demonstrated by synchrotron radiation small-angle X-ray scattering (SR-SAXS). Two-dimensional wide-angle X-ray diffraction (2D-WAXD) and Raman spectra were used to study the conformational changes in the obtained silk fibers. Furthermore, steered molecular dynamic simulations were performed to confirm the experimental results. Finally, the conducted strain–stress of the obtained silk fibers was measured.

## 2. Materials and Methods

Materials. Mulberry leaves and Bombyx mori silkworms were obtained from Zhejiang Sericulture Base Co., Ltd. (Zhejiang, China). Sodium alginate and nitric acid were purchased from Aladdin Industrial Co. Ltd. (Shanghai, China), sodium carbonate was purchased from Sinopharm Chemical Reagent Co., Ltd. (Beijing, China), and liquid metal (GaInSn, 15 °C) was purchased from Yunnan Zhongxuan Liquidmetaltechnology Co., Ltd. (Yunnan, China). All the solutions were prepared using ultrapure water. Also, all the used chemical agents in this study were of analytical grade, and they were received without further purification.

Characterization. The transmission electron microscopy (TEM) images of the LM@NaAlg nanodroplets were studied using a JEM-2100 microscope (JEOL, Tokyo, Japan). The sizes of the LM@NaAlg nanodroplets were measured by Zetasizer Nano (Nano-ZS90). SEM and EDS characterizations were carried out using an FEI Quanta microscope operated at 20 kV and 10 kV, respectively. The collected cocoons were first boiled twice in 0.5% (*w*/*v*) NaHCO_3_ solution for 30 min for degumming and then rinsed with deionized water. The degummed silk fiber was dried thoroughly at room temperature. The silk fiber was ground into powder, potassium bromide was referenced according to the mass ratio of 1:100, and samples were prepared by tablet pressing. FTIR spectra were carried out using a Spectrum BX (PerkinElmer, Cambridge, UK) spectrometer ranging from 1800 to 600 cm^−1^. The protein secondary structure contents were calculated by performing Fourier deconvolution over the amide I region (1600–1700 cm^−1^) and by integrating the peak area. The 2D-WAXD portion was measured by a Hypix-6000 photon direct reading detector (HomeLab, Rigaku, Japan). The effective photosensitive area was 77.5 mm * 80.0 mm, the focal spot diameter of electron beam was 100 μm * 100 μm, the saturation photosensitive capacity of single frame was more than 5.9 * 1011 cps, and the reading time of single frame was 7.4 ms. XRD patterns were recorded using an X-ray diffractometer (D8 Advance, Bruker, Karlsruhe, Germany) with a Cu Kα radiation at (40 kV and 40 mA). The samples were scanned at 5°–60° with a step of 0.02° using a curved position-sensitive detector. The thermal stability of the silk fibers was measured using thermogravimetric analysis (TGA) from 30 to 800 °C in N_2_ (99.99%) at a scanning speed of 15 °C min^−1^. The strain–stress curves were measured using ten degummed silk fibers rubbed into a cluster that belongs to self-twist yarn structure. For the measurement, one end of the silk fibers was fixed, and the other end rotated at high speed to form a spiral self-twist yarn with a diameter of 0.2 mm and at an extension rate of 1.0 mm min^−1^ using a SHIMADZUAG-IS tensile tester. For the Raman measurements of the liquid metal nanodroplets in the silk fibers, 6 mg of degummed silk fibers were dissolved in 1 mL of concentrated nitric acid. The obtained solutions were diluted 20 times using deionized water and then treated using ultrasonication. The final Raman spectra were collected using laser confocal Raman microspectroscopy (Renishaw inVia, Gloucestershire, UK) with a 785 nm excitation laser.

Preparation of the LM@NaAlg nanodroplets. The LM@NaAlg nanodroplets were prepared according to the reported strategy but with a slight modification [36]. In brief, 20 mg of bulk LM were added to a 20 mL sodium alginate solution (0.6 wt%). The mixture was exposed to a probe sonicator (BILON92-II; power of 300 W with 80% amplitude) in an ice-water bath, thus preventing the reaction temperature from getting too high. The obtained suspension was washed with deionized water more than three times by centrifugation (5000 rpm, 20 min). Finally, LM@NaAlg nanodroplets were obtained with a diameter of ~40–250 nm.

Cytotoxicity test of the LM@NaAlg nanodroplets. A cytotoxicity test of the LM@NaAlg nanodroplets was performed using classic 3-(4,5-dimethylthiazol-2-yl)-2-diphenylte-trazolium bromide (MTT) assay on living human cervical carcinoma HeLa cancer cells and mouse breast cancer 4T1 cells. In detail, ~5 k living cells were cultured on a 96-well plate with a fresh DMEM medium (100.0 μL, 10% FBS, 1% penicillin-streptomycin) for 24 h in dark. Afterward, the culturing mediums were carefully replaced with FBS-free DMEM with a series of various concentrations (0, 0.01, 0.1, 0.5, 1.0, 2.0, 5.0, 10.0, 50.0, and 100.0 μg mL^−1^) of LM@NaAlg nanodroplets added to each well for a further 24 h and 48 h of incubation at 37 °C. In this case, 50 μL of MTT (1 mg mL^−1^) were also added to each well for another 4 h of incubation at 37 °C. Finally, the culturing medium in each well was removed, and 150 μL of DMSO were further added into each well, followed by about 20 min of shaking, thus achieving full dissolution of the produced blue formazan. Finally, the cell viability with a ratio of the absorbance at 490 nm of the pretreated and treated cells was measured by ultramicro-orifice spectrophotometer (BioTek, EPOCH2). 

Silkworm feeding. The silkworm larvae were fed with fresh mulberry leaves until the first day of their fifth instar. At the early stage of the fifth instar, the cellular structures for the synthesis of fibroin were rapidly formed, and the synthesis of fibroin proceeded at a maximum rate at the later stage, which was the key to rationally using mulberry leaves and improving the quality of silk fibers. Then, they were divided into four groups, each containing 20 silkworms with similar body weights. From the second day of the fifth instar, three experimental groups were fed with mulberry leaves sprayed with an LM@NaAlg solution concentration of 1, 2, and 3 wt%, named LM@NaAlg-1, LM@NaAlg-2, and LM@NaAlg-3, respectively. The silkworms fed with natural mulberry leaves sprayed with 0.6 wt% sodium alginate were designated as the control group. In each group, 5 mL LM@NaAlg nanodroplets aqueous solution was sprayed on 40 g mulberry leaves and fed every 24 h. The control group was sprayed with sodium alginate solution in the same method and dose. The silkworms fed with natural mulberry leaves sprayed with 0.6 wt% sodium alginate were designated as the control group. The temperature (25 °C) and humidity (65–75%) were carefully controlled in different growth periods.

Cocoon degumming. The obtained cocoons were dried at 110 °C for 90 min and at 75 °C for 180 min in a vacuum-drying oven and then the silkworm chrysalises were removed. Holes were cut in the cocoons, and they were boiled three times in an aqueous solution of Na_2_CO_3_ for 30 min before being rinsed thoroughly with deionized water. Finally, the collected degummed silks were dried at 70 °C in an oven for 6 h.

Steered dynamics simulation. In order to simulate shear conditions, steered molecular dynamic (SMD) simulations were performed in Amber99sb-ildn force field coupled with TIP3P water model by GROMACS, a free software available under the GNU Lesser General Public License (LGPL). Our protein sequence in National Center for Biotechnology Information database (SVISRAWDYVDDTDKSIAILNVQEILKDMASQGDYASQASAVAQTAGIIAHLSAGIPGDACAAANV) imported the protein sequence into SWISS-MODEL (https://swissmodel.expasy.org/, accessed on 14 March 2022), exported pdb file, and then constructed the topology in Amber99sb-ildn force field. Soft springs were fixed to the symmetry points of the equilibrated structures (residue TYR35). The springs were set to provide stiffness only in the pulling direction so that the structure was able to relax in all other directions, and hence no unnecessary constraints were enforced. An isothermal ensemble in an explicitly solvated periodic water box was set up at 298.15 K. Isobaric constraints were enforced in the two directions perpendicular to the pulling direction. The box size in the pulling direction was significantly larger than the molecule (7.8 × 7.0 × 17.5 nm^3^). For each run, the SHAKE algorithm and particle mesh Ewald method were used to determine bond lengths and long-range electrostatic interactions, respectively. A 12 Å cut-off was used for van der Waals forces and Coulomb interactions. The generated trajectories were evaluated using GROMACS inbuilt tools, and the results were visualized using VMD.

## 3. Results and Discussion

### 3.1. The Preparation of LM@NaAlg Nanodroplets and Effect on Silk Fibers

**Figure 1:**
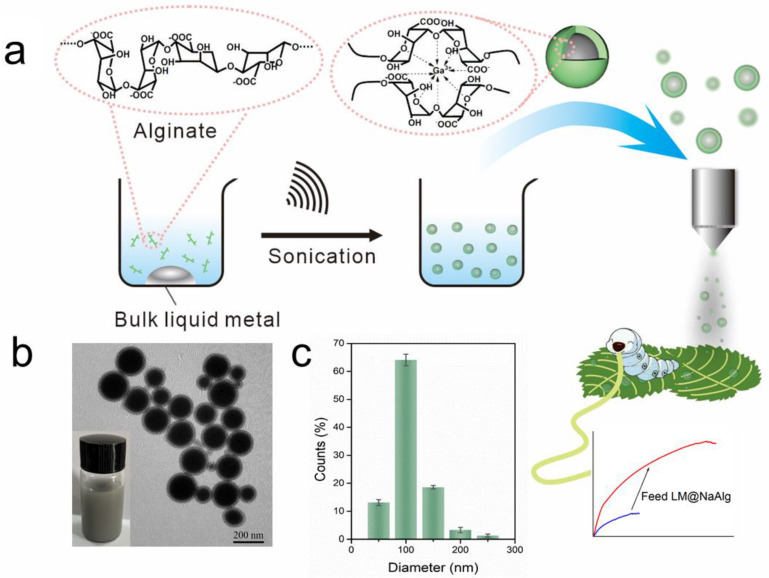
Preparation of the LM@NaAlg nanodroplet modified silks. (**a**) Schematic illustration of the sonicating liquid metal in the alginate solution and the liquid metal nanodroplets shelled in alginate microgel; (**b**) TEM image of the LM@NaAlg nanodroplets; (**c**) diameter histogram of the LM@NaAlg nanodroplets, sonication time of 60 min, and alginate concentration of 0.6 wt%.

**Figure 2:**
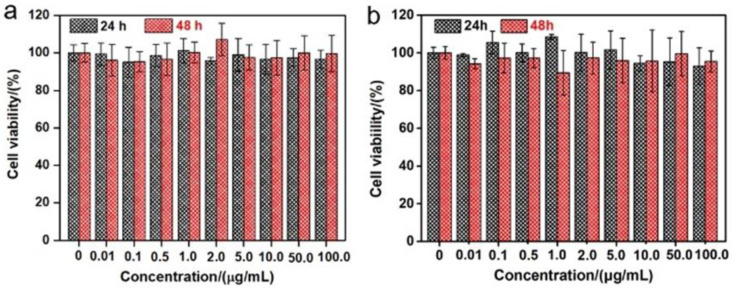
Cytotoxicity experiments of different concentrations of LM@NaAlg by using Hela cells (**a**) and 4T1 cells (**b**).

As shown in Figure 1a, aiming at hindering the rapid oxidation of the liquid metal when exposed to water and oxygen, at the same time, improving the biocompatibility, we first produced a stable aqueous ink of liquid metal by coating liquid metal nanodroplets with sodium alginate. It was reported that the carboxylic groups within the alginate G segments could chelate with multivalent cations, producing an “egg-box” structure and gelling alginate solutions [37]. The sodium alginate-protected liquid metal facilitated the downsizing process of liquid metal due to the coordination of their carboxyl groups with Ga^3+^. In this case, it formed microgel shells around liquid metal droplets by chelating Ga^3+^ into structural “egg-box” crosslinkers. After 60 min of sonication, a stable opaque slurry was obtained, and the LM@NaAlg nanodroplets were obtained after washing and size-grading with a diameter of 40–250 nm and an average value of 116 nm (Figure 1b,c). The thickness of the alginate layer coated on the surface of the liquid metal droplets was around 20 nm, which was measured from the TEM image of the LM@NaAlg (Figure 1b), and the uniformity of the sodium alginate layer was excellent, though the sizes of LM@NaAlg nanodroplets were not uniform. The LM@NaAlg nanodroplets were stable without coalescing and oxidizing for a long period of time of >7 d in the air due to their mechanical robustness and the decreasing oxygen permeability of the microgel shells. The stability of the LM@NaAlg nanodroplets was shown in Figure S1, and the TEM image measured after six days’ preservation continued to present excellent morphology with the undestroyed shell of sodium alginate. However, precipitation began to appear on day 8, and it was more obvious on day 10. Next, MTS assays were conducted to evaluate the cytotoxicity of the LM@NaAlg to HeLa cells and 4T1 cells. As shown in Figure 2, the Hela cell viability was estimated to be greater than 95% with 100 μg mL^−1^ of LM@NaAlg for 24 and 48 h, indicating the obtained remarkable biocompatibility. After being cultivated with different concentrations of LM@NaAlg nanodroplets for 24 h and 48 h, 4T1 cells were in good shape, but a negligible decrease could be observed when the 4T1 cells were incubated for 48 h, potentially attributed to the excessive consumption of the culture medium. Overall, the significant biocompatibility of the LM@NaAlg paved the way to produce stretchable silk as the diet of silkworms.

### 3.2. Effect of LM@NaAlg in Feeding Diet on Growth and Silk of Silkworms

**Figure 3:**
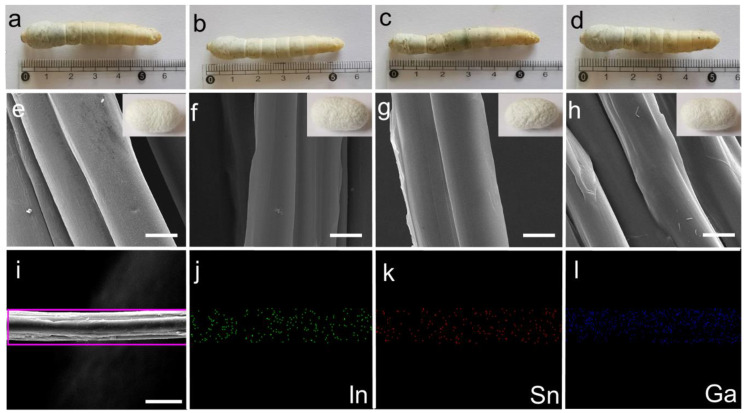
(**a**–**d**) Photographs of the mature larvae and vignettes of cocoons from the silkworms fed with different diets in the order of Control, LM@NaAlg-1, LM@NaAlg-2, and LM@NaAlg-3, showing no observable difference. (**e**–**h**) SEM images showing the morphology of the degummed silk fibers corresponding to the cocoons shown in (**a**–**d**). (**i**–**l**) EDS mapping of the selected area of the silk fibers. The scale bar is 10 μm.

We separated 80 silkworms into four groups, each containing 20 silkworms with similar body weights. Three experimental groups were fed with mulberry leaves sprayed with an LM@NaAlg solution concentration of 1, 2, and 3 wt%, named LM@NaAlg-1, LM@NaAlg-2, LM@NaAlg-3, respectively. No obvious differences between the silkworms fed with different diets were observed until cocoons were produced, demonstrating that the diets containing the used LM@NaAlg nanodroplets in this work were safe for raising silkworms (Figure 3a–d). Also, the photographs of the as-obtained cocoons shown in vignettes exhibited similar colors and uniform sizes. The average silkworm weight mass was recorded 7 days after feeding the additives of the LM@NaAlg nanodroplets (Figure S2), and all the silkworms had similar weights over the 7 days duration of the fifth instar with no obvious differences between those fed with the LM@NaAlg nanodroplets diet and the control groups. The silkworm larvae survival rate for each group demonstrated that the LM@NaAlg had a negligible effect with a mortality rate of 0.05% for the LM@NaAlg-2 and LM@NaAlg-3 groups (Table S1). This demonstrated that the modified diets in our work were safe for silkworms. In addition, the cocooning rate, cocoon shell weight, and silk diameter were also studied (Figures S3–S5) to further investigate the security of the used LM@NaAlg nanodroplets. The cocooning rate was slightly different due to the different uptake of the various LM@NaAlg concentrations. The biggest silk diameter (2 wt% group) may be attributed to the high breaking elongation data, and the SEM image of silk fiber cross-sectional shapes is shown in Figure S6. This can be considered the optimal concentration for the LM@NaAlg nanodroplets uptake in our work.

All the silk cocoons were degummed to remove the sericin coating on the silk fibers, so they could be used in the following characterization. The diameter and morphology of the degummed silks were characterized using an SEM. As shown in Figure 2e–h, we found that the silks generated from LM@Alg-1, LM@Alg-2, and LM@Alg-3 exhibited similar morphology compared with the control group, indicating that the incorporation of LM@NaAlg nanodroplets did not have an apparent influence on the silk morphology. The results of the energy-dispersive spectra (EDS) mapping of the modified silk displayed the uniform distribution of the content and the distribution of the gallium (Ga), indium (In), and stannum (Sn) elements in the silk fiber (Figure 2i–l), indicating that LM@NaAlg perfectly combined with the silks. The content distributions of the different elements in the modified silk fibers were further investigated. Ga (1.31 wt%), In (0.46 wt%), and Sn (0.42 wt%) were obviously detected, while C (60.19 wt%) and O (34.91 wt%) were dominant, indicating the successful combination of liquid metal and silk fibers (Figure S7 and Table S2).

### 3.3. Thermal Degradation of Degummed Silks

The thermal stability of the modified silk fibers was measured using thermogravimetric analysis (TGA) under a nitrogen atmosphere at a scanning speed of 15 °C min^−1^. The results of the TGA and differential thermos gravimetric (DTG) curves showed the mass change in the modified silk fibers during the heating process from 30 °C to 800 °C (Figure S7). The intermolecular-bound residual water in the silk fibers was removed as the temperature increased to 150 °C, and the silk fibers exhibited similar thermal degradation curves with a rapid mass decrement from around 295 °C. Specific significant weight loss temperatures are shown in Figure S8. The DTG curves were the first derivatives of the corresponding TAG curves. When the silk was heated, the molecules in the amorphous region of the fiber were first moved. As the temperature increased, the molecular chain in the crystallization zone gradually moved, and the macromolecular chain was cleaved [34]. For the control group, the starting and highest decomposition temperatures of the decomposition were 291.58 °C and 329.81 °C, respectively. These decompositions occurred at higher corresponding temperatures for the LM@NaALg-1 group at 294.71 °C and 329.87 °C, respectively, for the LM@NaALg-2 group at 297.66 °C and 322.65 °C, respectively, and for the LM@NaALg-3 group at 295.25 °C and 348.4 °C, respectively, owing to the liquid metal nanodroplets in the silk fibers. The TGA and DTG results thus prove that the addition of liquid metal nanodroplets in the silk fibers using modified LM@NaAlg nanodroplet diets can enhance the thermal stability and slow the thermal degradation of silkworm silk.

### 3.4. Secondary Structure Characterizations of Degummed Silks

**Figure 4:**
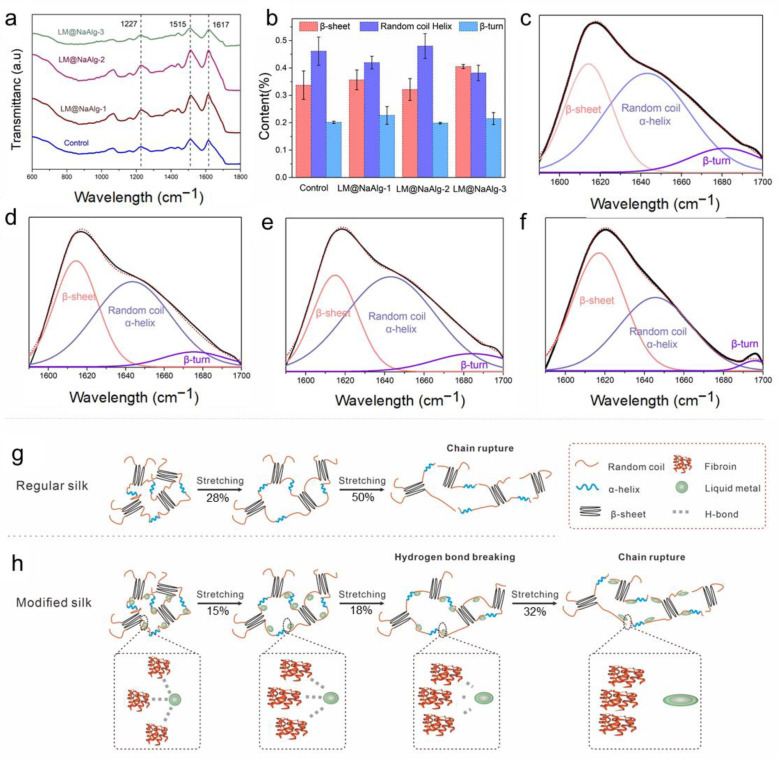
Effect of the LM@NaAlg nanodroplets on the secondary structures of the obtained silks. (**a**) FTIR spectra of the degummed silk fibroins of the silk samples; (**b**) contents of the secondary structures of the silk samples acquired according to the deconvoluted amide I band spectra; (**c**–**f**) deconvolution of the FTIR spectra in the amide I band of (**c**) Control, (**d**) LM@NaAlg-1, (**e**) LM@NaAlg-2, and (**f**) LM@NaAlg-3; (**g**,**h**) schematic illustration of regular silk and the interactions between the modified silk and LM@NaAlg nanodroplets.

Fourier-transform infrared spectroscopy (FTIR) is one of the most powerful methods for characterizing the second structure and super inter-interactions of silk fibers (Figure 4a) [11,34]. The signal at 1227 cm^−1^ was assigned to the β-sheets and random coils or/and α-helixes [38]. Also, the signal at 1617 cm^−1^ was considered to be attributed to the β sheet conformation, and the absorb peak at 1513 cm^−1^ was ascribed to the β-sheet structure due to the N-H deformation [38]. The identical peak position of the FTIR spectra confirmed that the LM@NaAlg nanodroplets did not have covalent interactions between the basic structure of the silk fibers and the LM@NaAlg nanodroplets. Fourier self-deconvolution (FSD) was conducted for the corresponding content of the amide I regions to quantify the β-sheet, random coils/α-helix, and β-turn contents to demonstrate the nano–bio interactions in the silk fibers (Figure 4b–f). The contents of the random coil and the helix of LM@NaAlg-1 were 0.41 and 0.38 in the LM@NaAlg-3 group, respectively, while the contents of the β-sheet were 0.35 and 0.4, respectively. Both were higher than the control group (0.33). The LM@NaAlg-modified silks contained a greater number of chains in the random coil/α-helix and β-turn conformations compared with the control silk. The β-sheet content of the control group was approximately 33.7%, which was better than that of the LM@NaAlg-2 group (32.1%). This may be ascribed to the abundant carboxyl and hydroxyl on the surface of LM@NaAlg nanodroplets, which were in favor of forming hydrogen bonds with the amino groups of silk fibers and slightly hindered the conformation transition of the silk protein from random coil/β-turn to β-sheet, corresponding to the better strength and stiffness. The proposed reinforcing schematic illustration shows the interactions between the LM@NaAlg nanodroplets and silk fibers (Figure 4g–h). During stretching, the random coil/α-helix conformational chains were the first to deform in the amorphous phase because they were easily movable. At the same time, the nanometer size scale, intensive hydrogen bond interactions, and spherical morphology caused the LM@NaAlg nanodroplets to move with the protein chains, providing more space for the chains to move, while about 28% and 15% of hydrogen bonds between protein–protein in the control and the LM@NaAlg-modified group were destroyed during the first stretching process. After 12 s, the proportion of it stood at 50% and 32%, respectively. This collaborative mobility promoted a larger elongation at the break to the modified silk fibers. Furthermore, the higher orientation and increased content of mesophase further enhanced the mechanical properties of the LM@NaAlg-modified silk fibers.

### 3.5. Steered Molecular Dynamics of LM@NaAlg-Modified Silk Fibers

**Figure 5:**
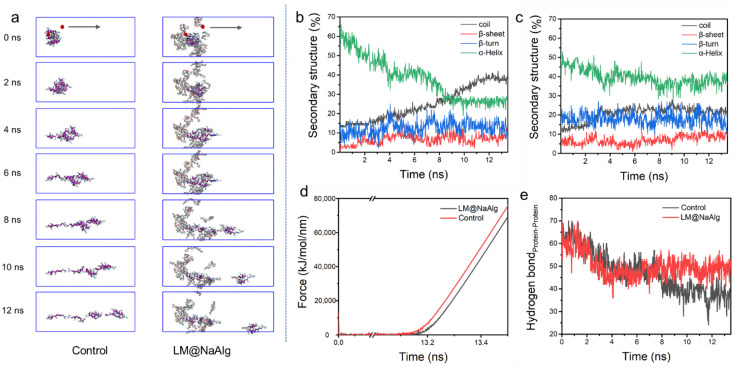
Steered dynamics simulation of the silk. (**a**) The pull process of the silk protein without and with LM@NaAlg-2. The secondary structure of silk protein in the pulling process without (**b**) or with (**c**) LM@NaAlg-2. (**d**) Shear stress associated with the secondary structure transition. (**e**) Hydrogen bond number between silk protein and LM@NaAlg-2 during the steered dynamics process.

Steered molecular dynamic (SMD) simulations are performed to further investigate the mechanical properties of the LM@NaAlg-modified silk fibers. The left end of the silk fiber was fixed and stretched the middle of the rest portion, shown in Figure 5a. In the process of silk stretching, the system was more easily broken up without LM@NaAlg, while it was relatively stable with LM@NaAlg; after 4s, the difference could be observed. The secondary structure of the silk fiber changed during the stretching process, as shown in Figure 5b,c, which further highlights the LM@NaAlg’s contribution to the stretchable silk. Figure 5d shows the change of tension with time. When LM@NaAlg exists, the system generates stress later and becomes more stable, which can essentially be attributed to the H-bond of the silk fiber shown in Figure 5e. The H-bond number of LM@NaAlg-contained silk was gradually increased, rose slightly, and then leveled off, but the control group was declined until the structure was completely destroyed. The statistics in Figure S9 can also prove this. The SMD simulations directly performed the stretching silk combined with LM@NaAlg.

### 3.6. Crystalline Structure Characterization of Silk Fibers

**Figure 6:**
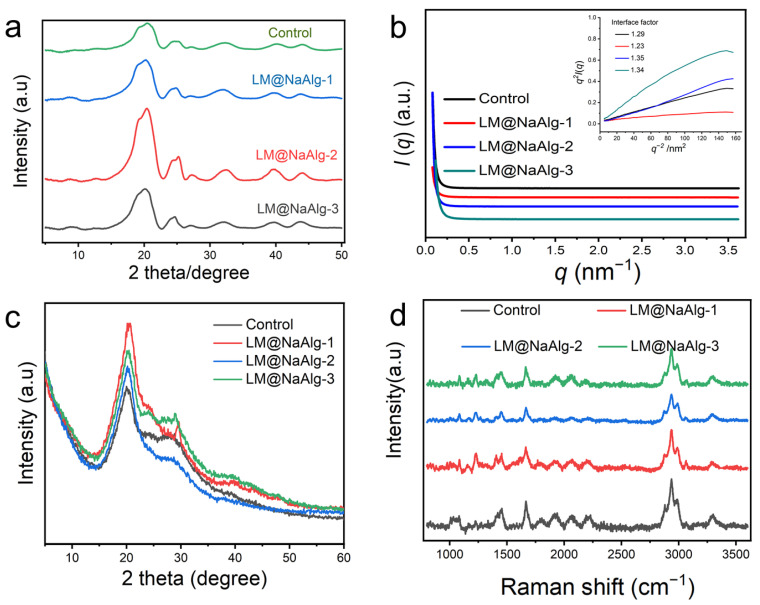
Crystalline structure characterization of silk fibers. (**a**) 1D WAXD of LM@NaAlg modified silk. (**b**) SR-SAXS patterns of degummed silks. The inset in panel b is the q2I(q)–q-2 curve based on the modified Porod formula. I(q) is the scattering intensity and q is the scattering vector. The interface factor is listed in panel (b). (**c**) XRD patterns of the silk fibers. Silk fibers are modified with different concentrations of LM@NaAlg nanodroplets and Control. (**d**) Raman spectra of the LM@NaAlg nanoparticles with different melting temperatures in the silk fibers of the silkworms fed with the diets that contained different concentrations of LM@NaAlg. The position of the characteristic peak was not significantly different between the groups.

It is essential to discuss the role of the interface phase, or mesophase, which acts as a modulus intermediate between the amorphous and crystalline phases and is of great significance in influencing the mechanical properties of silk fibers. To further characterize the crystalline structure of the silk fibers, 2D-WAXD and XRD were used, and the resultant patterns are shown in Figure 6. As seen from Figure 6a and Figure S10, the 1D and 2D WAXD patterns do not show obvious differences but differ in intensity. This is true for the highly stretchable silk, and mainly because of this the modification of LM@NaAlg impedes the conformation transition of silk fibroins from the random coil and α-helix to the β-sheet by the formation of hydrogen bonds between LM@NaAlg and the silk fibroins, thus enhancing the elongation at the breakpoints, in addition to the tensile properties. Hance increased the intensity of WAXD. Furthermore, the evidence for the mesophase in silks was also implied by SR-SAXD. The 2D SR-SAXD patterns were shown in Figure S11. Figure 6b illustrates the 1D SR-SAXD spectra of the silks. The inset pattern was the profiles of q2I(q)−q-2 based on the modified Porod law, which was used to calculate the interface factor. The interface thickness was approximately calculated and is listed in Table S3 [39]. The inset of Figure 6b suggests that the interface factors of the modified silks are larger than that of the control group. Since the interface factor was in direct proportion to the thickness of the interface, it is rational to consider that LM@NaAlg-modified silks have a thicker interface than control silk. The thickness of the interface increased to the highest figure with LM@NaAlg-2. However, when the concentration increased to LM@NaAlg-3 or declined to LM@NaAlg-1, the thickness of the interface decreased a little, which might be attributed to the poorer combination between silk fibroin and LM@NaAlg.

All the XRD patterns showed two typical diffraction peaks at around 20.5° (Figure 6c), which were attributed to the characteristic peaks of the β-sheet crystalline structure [40,41]. There was no noted difference among these patterns, regardless of the LM@NaAlg nanodroplet concentrations in each group. This phenomenon was consistent with the results of FTIR, indicating that the basic structures of silk fibers were barely changed by feeding LM@NaAlg nanodroplets. 

To confirm the conformational changes in the obtained silks upon the intake of LM@NaAlg in the silk cocoons, Raman spectroscopy was performed (Figure 6d). For the silks from either the LM@NaAlg or control groups, the most prominent Raman-active bands were observed at the same positions. The peak at 1,084 cm^−1^ represents the random coil conformation, and the peak at 1231 cm^−1^ could be assigned to the predominantly β-sheet conformation. The observed peak at 1666 cm^−1^ corresponds to the β-sheet/β-turn conformation, and the peaks at 2936 cm^−1^ and 3986 cm^−1^ correspond to the CH_3_ asymmetric stretch and N-H stretch, respectively [41]. In general, a similar intensity ratio and peak position were obtained, indicating that the conformation of the silks barely changed with the intake of the LM@NaAlg nanodroplets.

### 3.7. Stretchable Mechanical Properties of LM@NaAlg-Modified Silks

**Figure 7:**
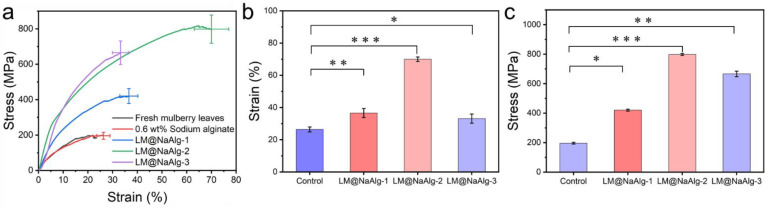
Typical stress–strain curves of the silk fibers. (**a**) Mechanical properties of the silk fibers modified with different concentrations of LM@NaAlg nanodroplets; (**b**) average stress histogram of control, LM@NaAlg-1, LM@NaAlg-2, and LM@NaAlg-3; (**c**) average strain histogram of control, LM@NaAlg-1, LM@NaAlg-2, and LM@NaAlg-3. Error bars represent standard deviations of three repetitive experiments (*n* = 3). *p* < 0.05: *; *p* < 0.01: **; and *p* < 0.001: ***.

The mechanical properties, including the breaking strength and elongation at the breakpoints of the silks, were closely associated with their secondary structures [42,43]. The typical stress–strain curves of the silk fibers shown in Figure 7 demonstrate the changes in the mechanical properties of the modified silks. It was observed that the LM@NaAlg-modified silks had significantly improved tensile properties compared with the control silks. The LM@NaAlg-1 silks exhibited a breaking strength of 420.43 MPa and elongation at a break of 36.58%. Also, the LM@NaAlg-3 silks possessed a breaking strength of 665.13 MPa and elongation at a break of 33.2%, considerably exceeding the values of control silks (196.14 MPa, 26.3%). When the silkworms were fed with an LM@NaAlg solution concentration of 2 wt%, a breaking elongation of up to 70% and a breaking strength of 814 MPa were realized, which are the highest values obtained so far (Table 1). As shown in Figure 7b,c, the average strain and stress measurements demonstrate the same trend that was accepted from the three-time measurement of the silk fiber in different groups. The remarkable improvement in the mechanical properties may be attributed to the excellent ductility of liquid metal and the high biocompatibility of LM@NaAlg nanodroplets. However, the high content of LM@NaAlg may aggregate and act as a defect, causing a low breaking strength or elongation at break.

## 4. Conclusions

In this work, we demonstrated that intrinsically reinforced silks can be simply produced by feeding silkworms with LM@NaAlg. Compared with control silk, the breaking strength and breaking elongation of the treated LM@NaAlg silk reached up to 814 MPa and 70%, respectively, which is the highest reported performance so far. This might be attributed to the formation of hydrogen bonds between LM@NaAlg and silk fibroin, leading to increased random coil/α-helix structures, higher orientation, and fewer β-sheet structures. In addition, we also examined the incorporation of LM@NaAlg, and it appeared to have a negative influence on the crystalline structure and conformation of silk fibers. Overall, this work provided a straightforward and effective way of improving the mechanical performance of silk. It might also shed light on the in vivo study of the liquid metal transportation mechanism and facilitate the large-scale production of reinforced silks. It is worth noting that several questions exist in our current investigation. For example, how to quantify and improve the efficiency of the LM@NaAlg uptake by the silkworms, how LM@NaAlg affects the silk structures in biological processes, and how LM@NaAlg is transported from mid-gut, hemolymph, and the silk gland into the silk. We also expect that researchers may be inspired to perform bio-interactions between functionalized nanomaterials and silkworms on the molecular, cellular, tissue, and even organ levels.

## Figures and Tables

**Table 1:** Summary of the mechanical property control-relative ratios of the silk fibers modified with different materials.

Sample	Breaking Strength Ratio	Breaking Elongation Ratio	Young’s Modulus Ratio	Reference
Liquid metal	4.06	2.5	1.25	The present study
Cu	1.3	1	1.2	[23]
Ag	1.2	1	1.1	[23]
MW CNT	2.1	1.4	2.7	[44]
SW CNT	0.8–1.7	0.6–1.3	1.0–1.2	[25]
Graphene	0.8–1.6	0.4–1.1	1.1–1.5	[25]
TiO_2_	0.6–1.4	0.4–1.4	0.9–1.5	[12]
Fe	1.5	1.25	1.08	[23]
CNTS	3.5	2.3	5.42	[45]
GQDs	1.4	1.2	1.7	[14]
Ca^2+^PO_4_^3−^	1.4	0.83	1.86	[46]
HPMC-MoO_2_	1.29	1.36	1.66	[47]
CNF	1.8	1.26	2.1	[48]

## Data Availability

Data is contained within the article.

## Supplementary Materials

The following are available online at https://www.mdpi.com/article/10.3390/nano12071177/s1, Figure S1: Stability test and TEM image of LM@NaAlg nanodroplets; Figure S2: The average weight of the silkworms fed with different diets from the second day of the fifth instar to the last 7 days; Figure S3: The silkworm cocooning rate of the different silkworms fed with different diets; Figure S4: The cocoon shell weight of the different silkworms in different groups; Figure S5: The silk diameter after removing sericin from the different groups; Figure S6: SEM and EDS of the modified silk fiber; Figure S7: TGA and DTG curves of the silk fibers of the Control, LM@NaAlg-1, LM@NaAlg-2, and LM@NaAlg-3 groups, respectively; Figure S8: The significant weight loss temperature of Control, LM@NaAlg-1, LM@NaAlg-2 and LM@NaAlg-3; Figure S9: The number of hydrogen bond with or without LM@NaAlg; Figure S10: 2D-WAXD of the degummed silks; Figure S11: 2D SR-SAXS patterns of degummed silks; Table S1: Silkworm larvae survival rate for each group; Table S2: Content distribution of different elements in the modified silk fibers; Table S3: Interface thickness (R) of the degummed silk.
