# Supplementary material for: Feeding Alginate-Coated Liquid Metal Nanodroplets to Silkworms for Highly Stretchable Silk Fibers
Source: Nanomaterials (Basel). 2022 Apr 1;12(7):1177. doi: 10.3390/nano12071177 (PMC9000898; doi:10.3390/nano12071177)
Supplement: Supplementary file 1 [file nanomaterials-12-01177-s001.zip › nanomaterials-1660310-supplementary.pdf]

Supplementary Information

Feeding Alginate-Coated Liquid Metal Nanodroplets to Silkworms for Highly Stretchable Silk Fibers

Zhong-Feng Gao [1,2,*], Lin-Lin Zheng [2], Wen-Long Fu [1], Lei Zhang [3], Jin-Ze Li [2] and Pu Chen [1,3,*]

[1] Advanced Materials Institute, Shandong Academy of Sciences, Qilu University of Technology, Jinan 250014, China; f58445646@163.com

[2] College of Chemistry and Chemical Engineering, Linyi University, Linyi 276005, China; zhenglinlinya@163.com (L.−L.Z.); ljz9711@163.com (J.-Z.L.)

[3] Department of Chemical Engineering and Waterloo Institute for Nanotechnology, University of Waterloo, 200 University Avenue West, Waterloo, ON N2L3G1, Canada; l78zhang@uwaterloo.ca

* Correspondence: gaozhongfeng@lyu.edu.cn (Z.−F.G.); p4chen@uwaterloo.ca (P.C.)

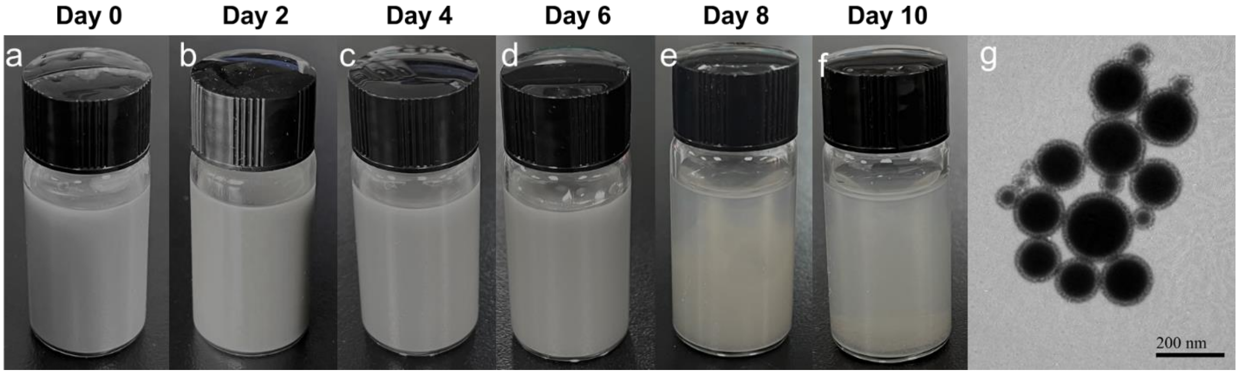

Figure S1. (**a–f**) Stability of LM@NaAlg nanodroplets. (**g**) Transmission electron microscopy image of LM@NaAlg nanodroplets after 6 days preservation.

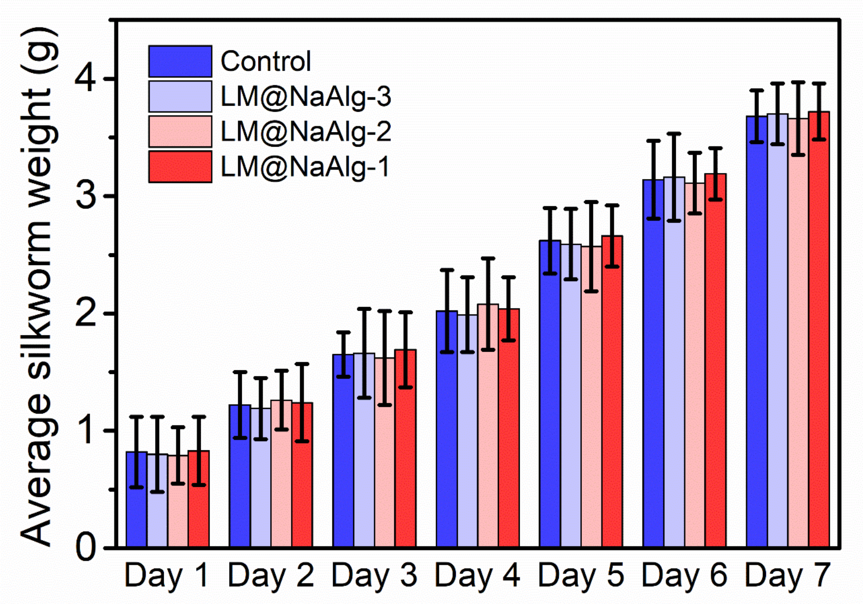

Figure S2. The average weight of the silkworms fed with different diets from the second day of the fifth instar to the last 7 days.

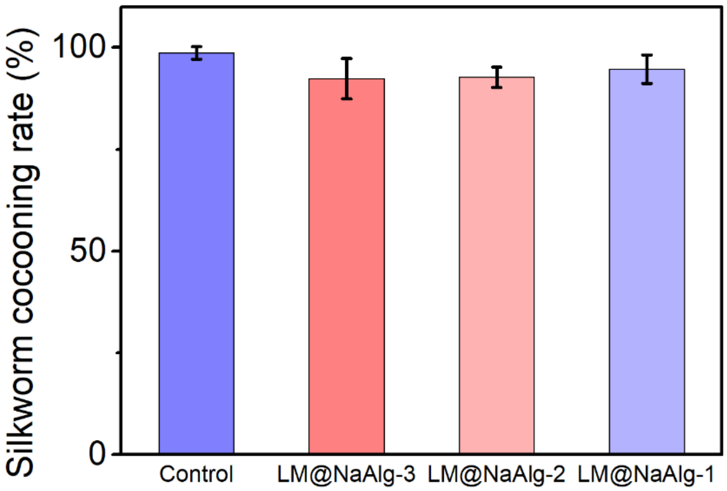

Figure S3. The silkworm cocooning rate of the different silkworms fed with different diets.

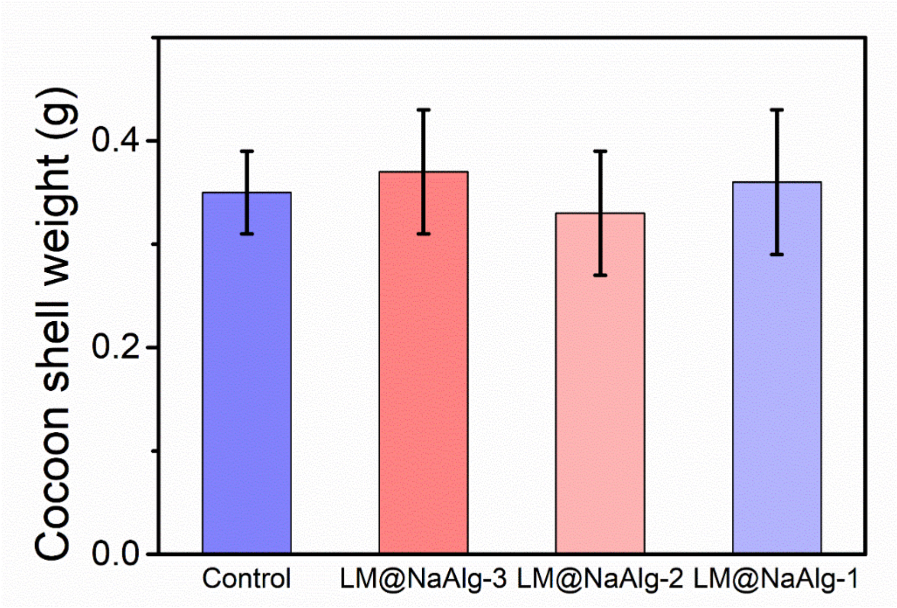

Figure S4. The cocoon shell weight of the different silkworms in different groups.

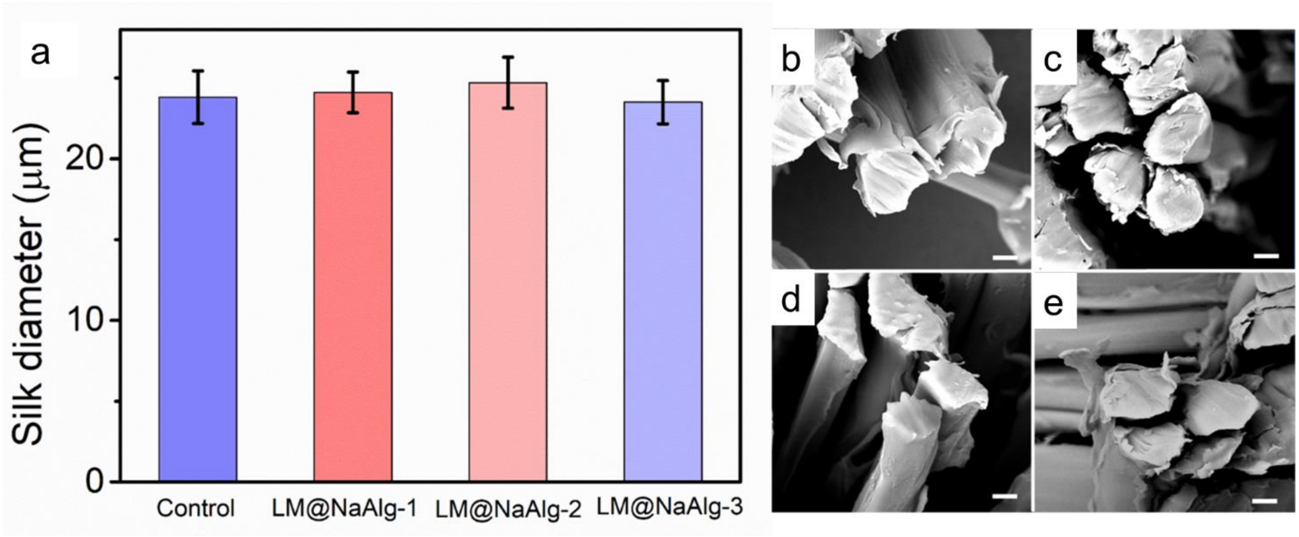

Figure S5. The silk diameter after removing sericin from the different groups. (**a**) Average diameter of single silk fiber. The silk fiber cross sectional shapes of (**b**) Control, (**c**) LM@NaAlg-1, (**d**) LM@NaAlg-2, and (**e**) LM@NaAlg-3 groups, respectively. The scale bar is 10 μm.

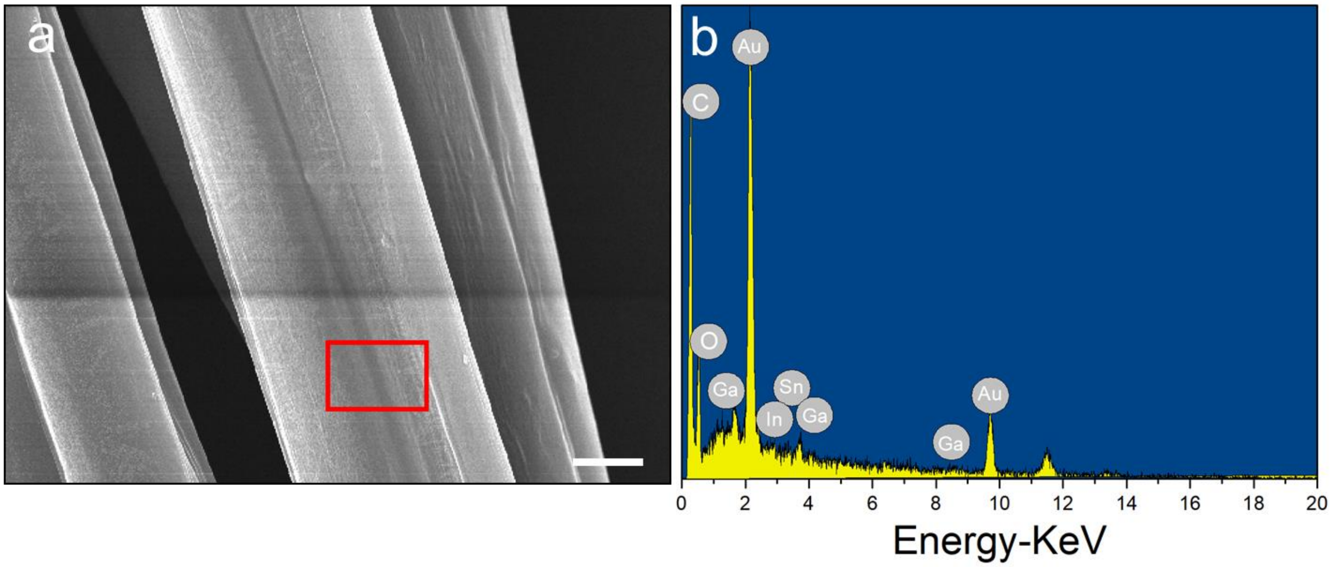

Figure S6. SEM of the modified silk fiber (**a**) and (**b**) the corresponding energy dispersive spectra (EDS) in the red square of LM@NaAlg-2. The high content of gold was due to the need to spray gold to improve electrical conductivity. The scale bar is 10 μm

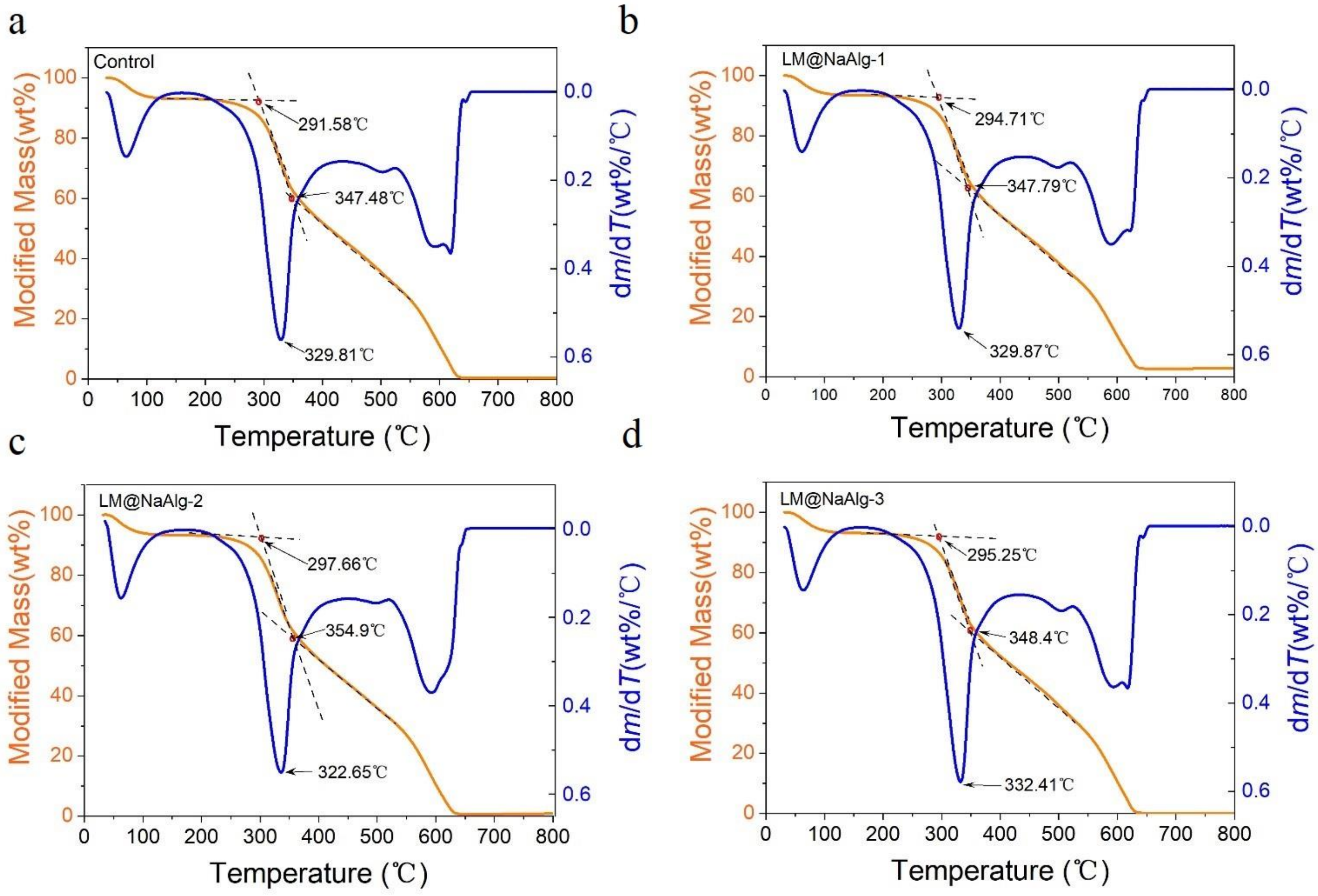

Figure S7. TGA and DTG curves of the silk fibers of the (**a**) Control, (**b**) LM@NaAlg-1, (**c**) LM@NaAlg-2, and (**d**) LM@NaAlg-3 groups, respectively. The blue and orange lines depict the TG and DTG curves, respectively, with a scanning speed of 15 °C min^{-1}.

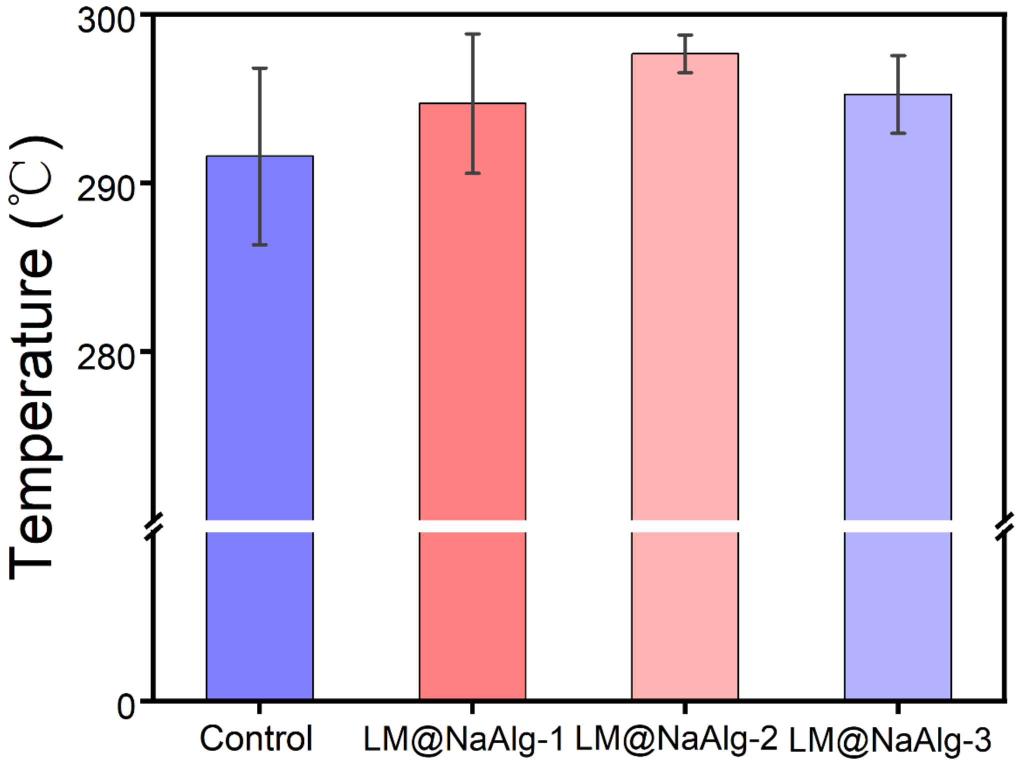

Figure S8. The significant weight loss temperature of Control, LM@NaAlg-1, LM@NaAlg-2 and LM@NaAlg-3.

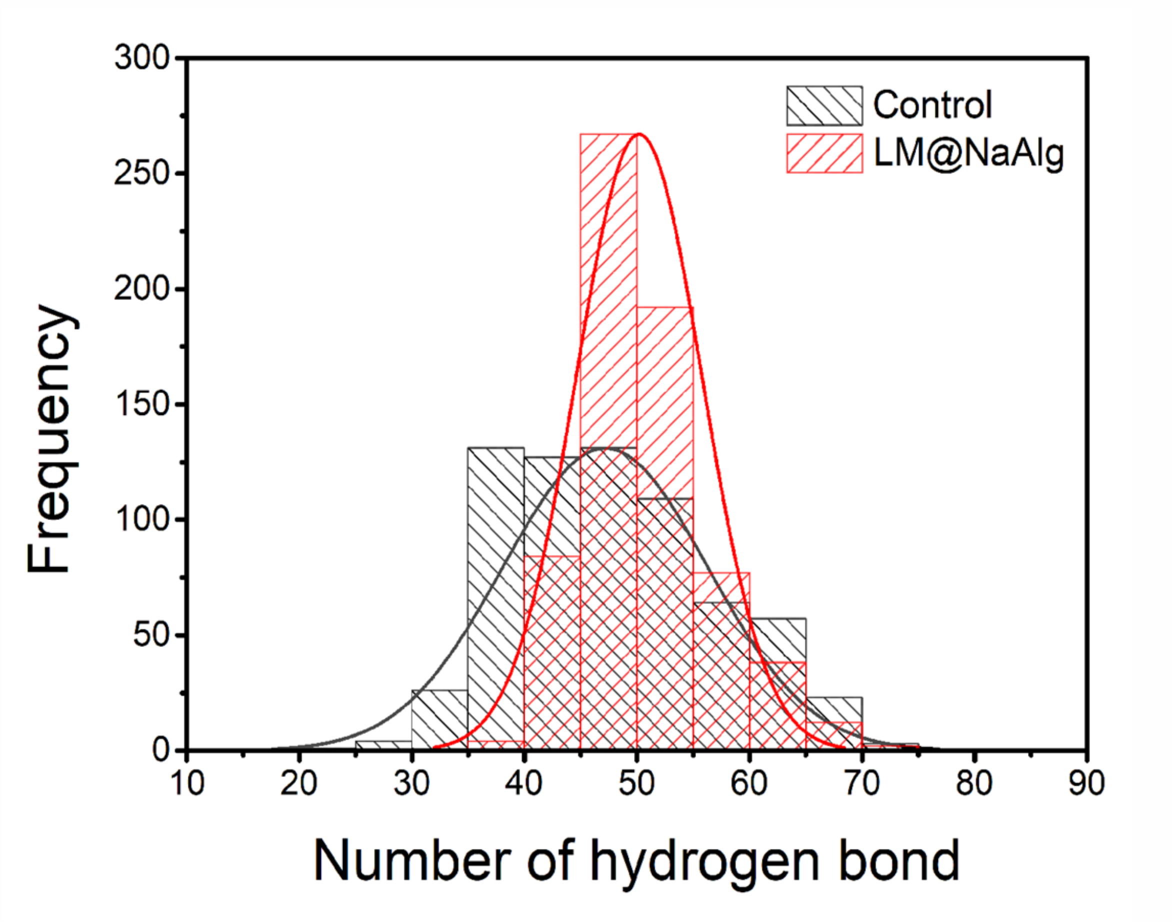

Figure S9. The number of hydrogen bond with or without LM@NaAlg.

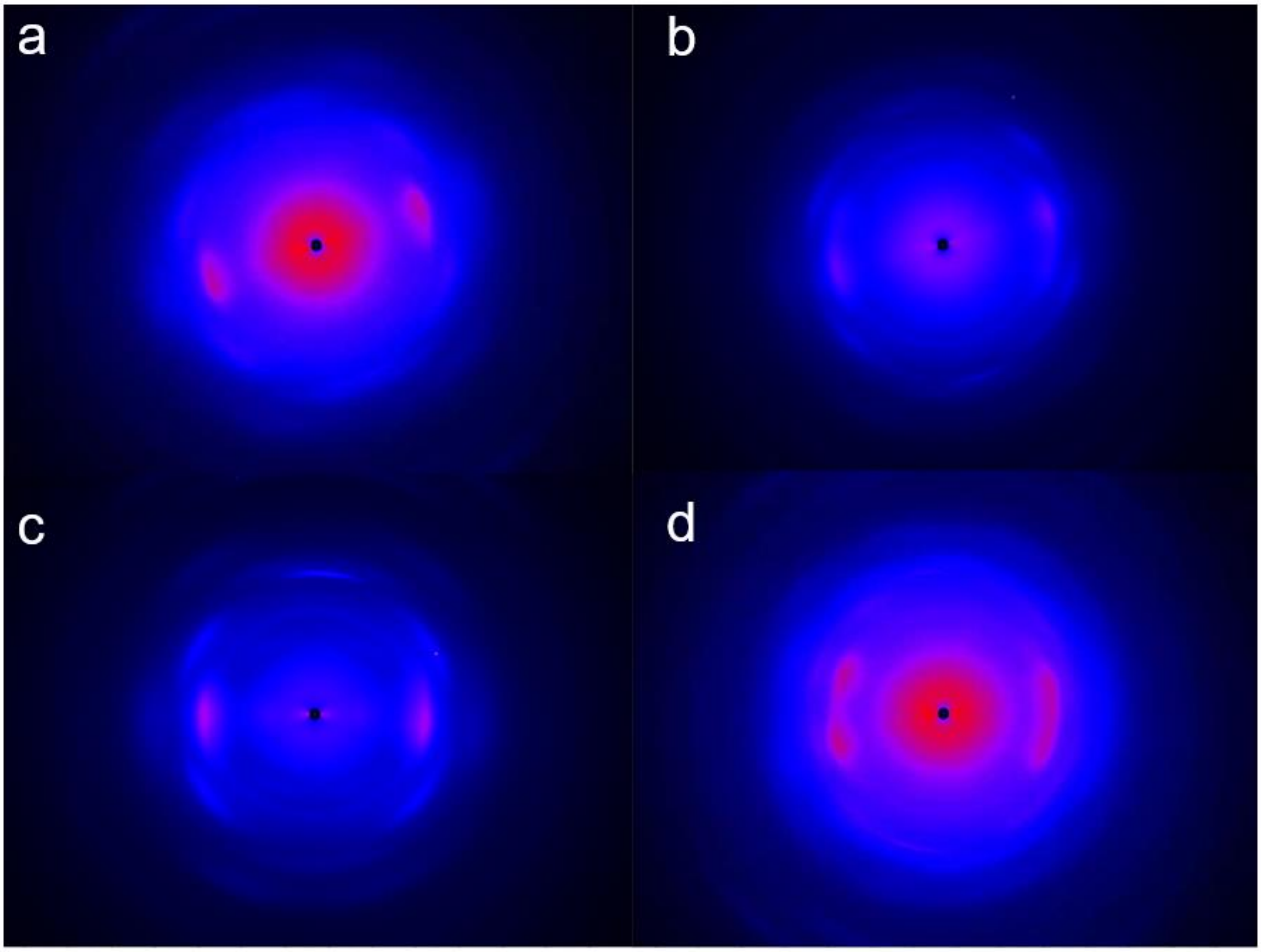

Figure S10. 2D-WAXD of the degummed silks. (**a**) Control group. (**b**) LM@NaAlg-1. (**c**) LM@NaAlg-2. (**d**) LM@NaAlg-3.

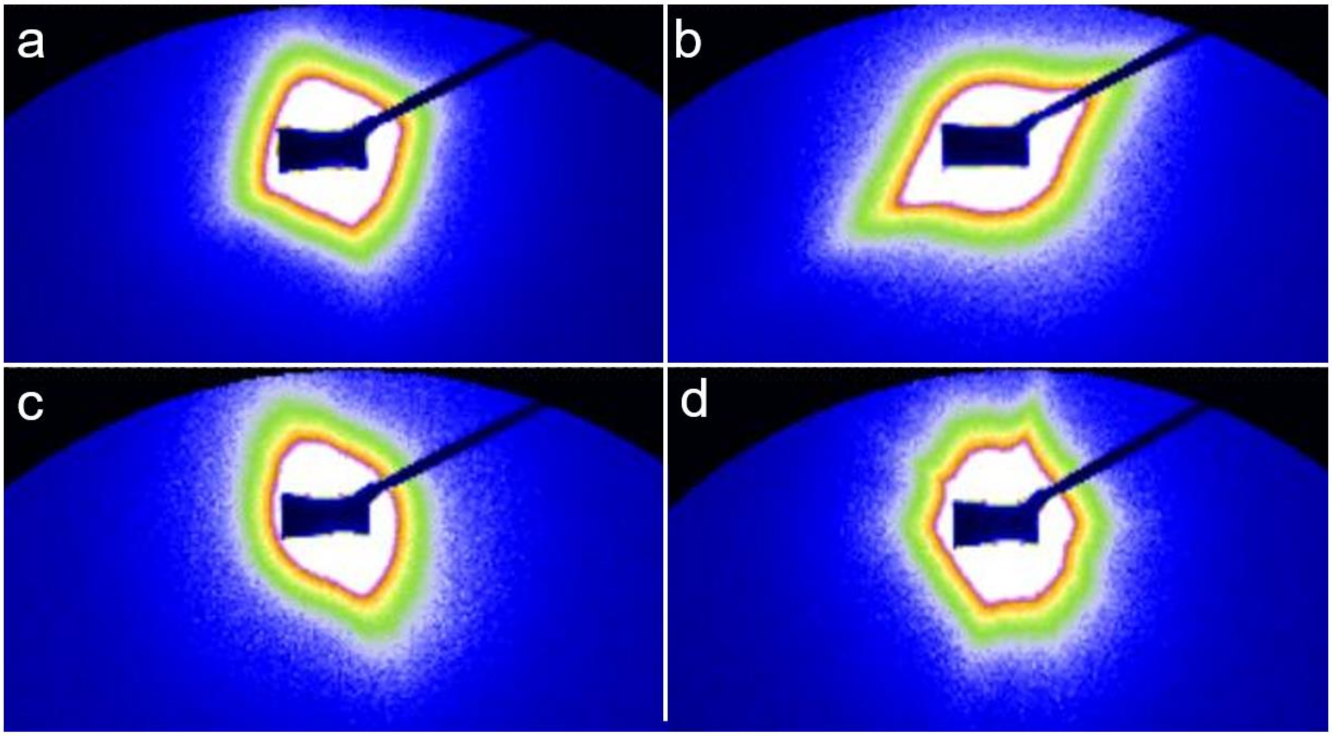

Figure S11. 2D SR-SAXS patterns of degummed silks. (**a**) Control group. (**b**) LM@NaAlg-1. (**c**) LM@NaAlg-2. (**d**) LM@NaAlg-3.

Table S1. Silkworm larvae survival rate for each group. N represents to the number of silkworms reared for each group, SN represents to survival number for each group without or with the intake of LM@NaAlg, MR represents to mortality rate.

**Sample**	**N**	**SN**	**MR (%)**
Mulberry leaves only	20	20	0
Sodium alginate	20	20	0
LM@NaAlg-1	20	20	0
LM@NaAlg-2	20	19	0.05
LM@NaAlg-3	20	19	0.05

Table S2. Content distribution of different elements in the modified silk fibers. In (0.46 wt%), Sn (0.42 wt%), and Ga (1.31 wt%) were obviously detected with the dominant C (60.19 wt%) and O (34.91 wt%), indicating the successful combination of liquid metal and silk fibers.

**Element**	**Wt%**	**At%**
CK	60.19	68.92
OK	34.91	30.01
InL	01.46	00.18
SnL	00.42	00.05
CaK	01.71	00.59
GaK	01.31	00.26
Matrix	Cottection	ZAF

Table S3 Interface thickness (ΔR) of the degummed silk.

**Sample**	**Control**	**LM@NaAlg-1**	**LM@NaAlg-2**	**LM@NaAlg-3**
ΔR (nm)	4.46	4.26	4.67	4.64
